# Outlet Ventricular Septal Defect in an Elderly Male

**DOI:** 10.7759/cureus.17127

**Published:** 2021-08-12

**Authors:** Gaurav K Sharma, Wasey Ali Yadullahi Mir, Daniela Kovacs, Zeina Ibrahim, Daniel Benatar, Sandeep Khosla, Suman Gaire, Dhan B Shrestha

**Affiliations:** 1 Cardiology, Rosalind Franklin University of Medicine and Science, Chicago, USA; 2 Internal Medicine, Mount Sinai Hospital, Chicago, USA; 3 Department of Emergency Medicine, Palpa Hospital, Palpa, NPL

**Keywords:** aortic valve insufficiency, congenital heart defects, hemodynamics, ventricular septal defects, adult

## Abstract

Ventricular septal defect (VSD) is the most common congenital cardiac anomaly in children and the second most common congenital cardiac anomaly in adults. The hemodynamic compromise associated with VSD is due to the shunt formation created by the abnormal communication between the right and left ventricles. While 85%-90% of small VSDs close spontaneously during the first year of life, some do not close spontaneously. If spontaneous closure does not occur during childhood, a VSD may persist into adulthood and may first be recognized after the development of a complication. We present a case of outlet VSD with secondary aortic insufficiency due to the prolapse of the aortic valve leaflet, especially in the right coronary cusp (RCC) sparing the left coronary cusp. RCC prolapse is an important finding in outlet VSD as the prolapse has the potential to cause permanent aortic insufficiency and closure is indicated regardless of the size of VSD.

## Introduction

Ventricular septal defect (VSD) is one of the most common congenital heart defects at birth but accounts for only 10% of congenital heart defects in adults [1,2]. Approximately 85%-90% of small VSDs close spontaneously during the first year of life, except for outlet VSD. It has been documented that the incidence of closure increases with age, from 24% at 18 months to 50% at 48 months, and 75% at 120 months [3]. If spontaneous closure does not occur during childhood, the VSD may persist into adulthood and be recognized after the development of a complication.

VSD is broadly classified into four major group types, namely, muscular or trabecular, inlet or atrioventricular canal, membranous, and infundibular or outlet VSD [4]. Membranous VSD is the most common type accounting for 80% of all defects, followed by muscular VSD accounting for 20% of all defects, and followed by inlet or atrioventricular canal VSD accounting for 8% of all defects [4]. The rarest form of VSD is the outlet VSD that only accounts for 6% of all VSD cases except for in the Asian population where it accounts for approximately 30% [4,5]. The main cause of hemodynamic dysfunction is the abnormal communication between the right and left ventricles leading to a shunt formation.

We present a case of an outlet VSD in a 61-year-old male with secondary aortic insufficiency due to prolapse of the aortic valve leaflet, especially in the right coronary cusp (RCC). RCC prolapse is an important finding in outlet VSD as the prolapse has the potential to cause permanent aortic insufficiency and closure is indicated regardless of the size of the VSD.

## Case presentation

A 61-year-old man from the Midwest United States presented with complaints of shortness of breath on exertion in the clinic. The patient had progressive worsening of shortness of breath for a couple of weeks. At the time of presentation, he felt breathlessness on walking a block distance. He did not complain of fever, chest pain, cough, shortness of breath at rest, or leg edema. He had a past medical history of diabetes mellitus, systemic and pulmonary hypertension, and chronic kidney disease. On examination, his vitals were stable. His cardiovascular examination was remarkable for a loud holosystolic murmur at the left lower sternal border. There were no signs of right- or left-sided heart failure. Other physical examinations findings, including neck, chest, and abdomen, were unremarkable. Blood reports were not significant.

**Figure 1:**
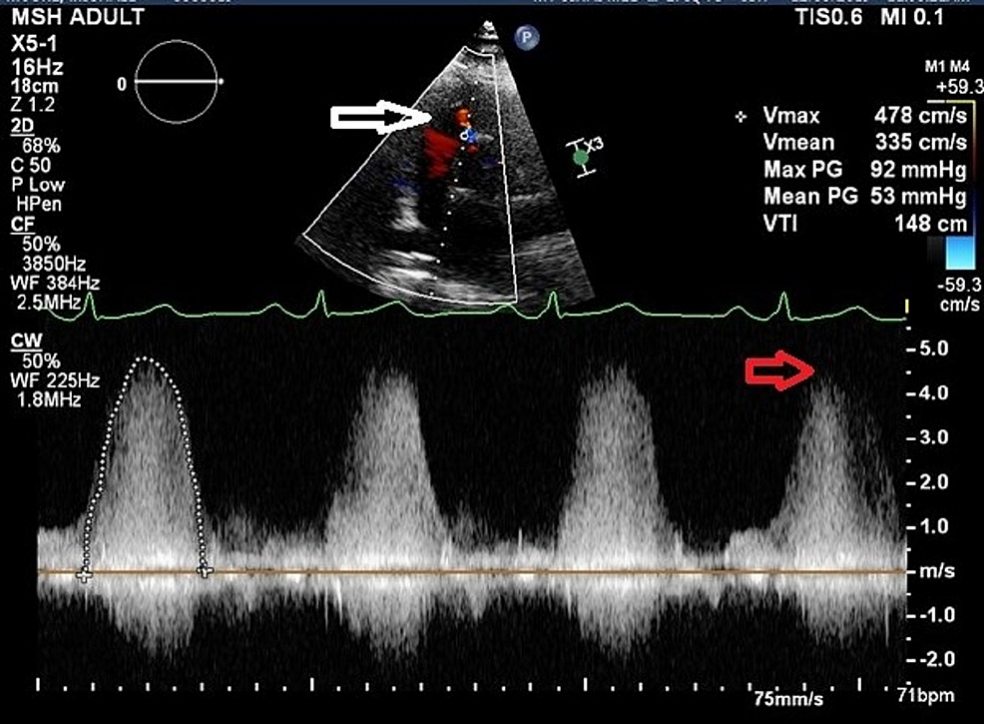
Transthoracic echocardiogram: VSD jet was identified to be around 4 m/sec, with the white arrow showing VSD and red arrow indicating velocity VSD: ventricular septal defect

**Figure 2:**
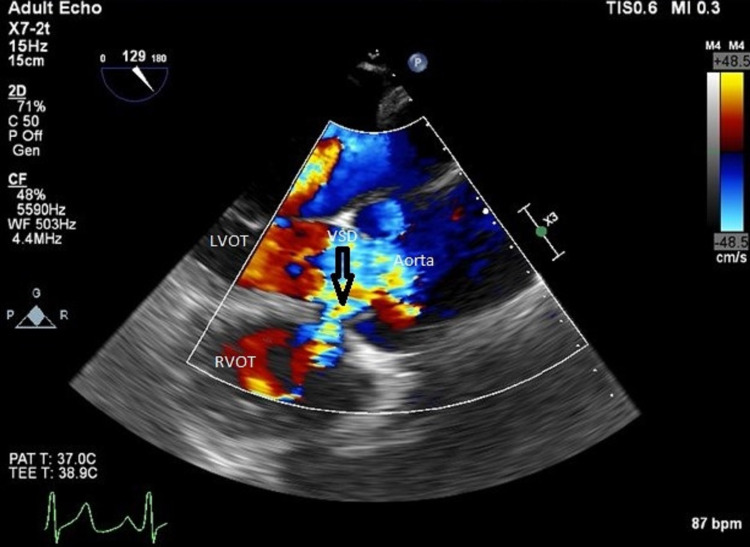
Transesophageal echocardiogram with color flow showing the left to right shunt (from the left ventricle to the right ventricle) through the outlet VSD LVOT: left ventricular outflow tract; RVOT: right ventricular outflow tract; VSD: ventricular septal defect

**Figure 3:**
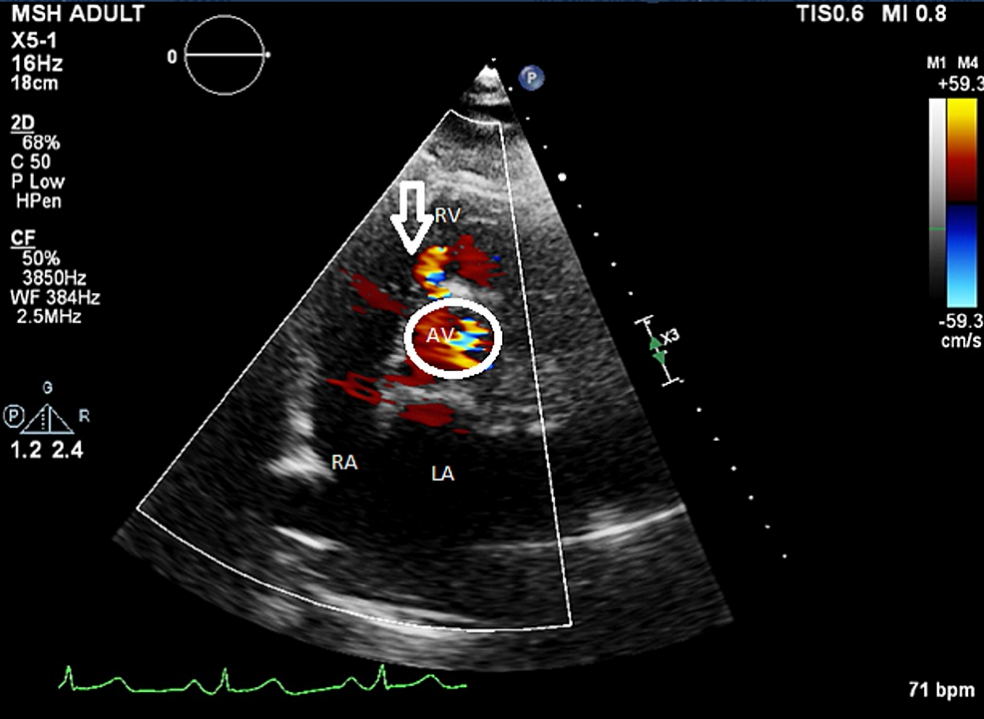
Transesophageal echocardiogram in the aortic valve short-axis view with the visible VSD jet at approximately 11 O’clock position VSD: ventricular septal defect; RA: right atrium; LA: left atrium; AV: aortic valve (circle) The arrow indicates the 11 O’clock position.

His baseline electrocardiogram and chest X-ray were normal. A transthoracic and transesophageal echocardiogram was performed. The transthoracic echocardiogram showed ventricular septal defect jet identified to be around 4 m/sec (Figure 1), which showed color flow across the interventricular septum in the apical five-chamber view and short-axis view concerning a VSD (Figures 2, 3). His ejection fraction was normal.

**Figure 4:**
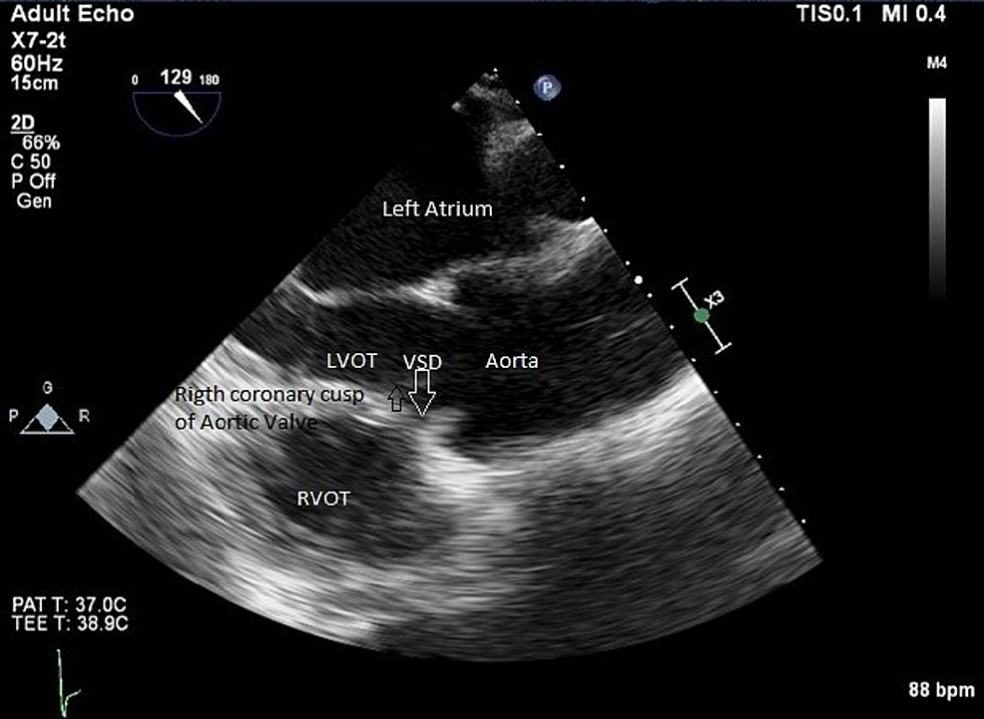
Transesophageal echocardiogram long-axis view of the aortic valve without color flow showing the left to right shunt through the outlet VSD and right coronary cusp prolapse VSD: ventricular septal defect; LVOT: left ventricular outflow tract; RVOT: right ventricular outflow tract The white arrow denotes VSD and the black arrow denotes the right coronary cusp of the aortic valve.

Transesophageal echocardiogram revealed a rare outlet VSD underneath the aortic valve. The defect was measured to be 7 mm in diameter. It was a restrictive VSD with a peak velocity of 4.5 m/sec, with a peak gradient of 81 mmHg (Figure 4).

Given the location of the VSD, prolapse of the right aortic valve coronary cusp into the left ventricular outflow tract during diastole causing secondary aortic regurgitation was noted. Moreover, he also had an aortic root dilatation that a cardiothoracic surgeon evaluated. The aortic root (measuring 5 cm at the sinus of Valsalva, which was indexed to body surface area [BSA] 2.2 cm/m^2^, at the sinotubular junction 4.1 cm with index to BSA 2 cm/m^2^ and measuring at proximal ascending aorta 3.8 cm indexed to BSA 1.8 cm/m^2^) was evaluated to complete the echocardiogram study. As the patient had the VSD causing prolapse of RCC and causing aortic regurgitation, which is an indication for surgery, a comprehensive evaluation was done. He was planned for the repair of the VSD using patch closure. The patient was also planned for aortic valve repair or replacement with a mechanical or bioprosthetic valve as per the patient's preference, along with aortic root replacement. He was referred for surgery to another center. The patient was then lost to follow-up.

## Discussion

We have presented a case of an outlet VSD complicated by secondary aortic regurgitation. The outlet VSD is located below the aortic and pulmonary valves in the outlet of the septum of the right ventricle. Secondary aortic insufficiency is a major complication of an outlet VSD in adults, and the prevalence of aortic regurgitation in patients with an outlet VSD increases with age [6]. In a retrospective study, 50% of patients had developed aortic regurgitation by 8 years of age, and by 20 years of age, 87% of the patients developed aortic regurgitation. With increasing access to health care facilities, most cases with an outlet VSD are detected earlier, and it has become increasingly rare to find such findings in the elderly population.

The outlet VSD leads to poor anatomical support for the coronary cusp, an important factor in causing aortic insufficiency [7]. The Qp/Qs ratio was 1.5 in our case, indicating a left to right shunt. Furthermore, the left to right shunt caused by VSD in early systole causes the prolapse of the right coronary cusp due to the venturi effect. During diastole, as the intra-aortic pressure causes the cusps to close, the unsupported cusp's free margin is further pushed down, leading to aortic insufficiency [8,9]. This explains the findings of the transesophageal echocardiogram in our patient.

The right coronary cusp prolapse is a significant finding in patients with an outlet VSD as the prolapse can cause permanent aortic insufficiency. Therefore, the patients with aortic regurgitation should undergo closure of the VSD regardless of the size [10-13]. Furthermore, the associated aortic valve defect should also be repaired as there is some risk of progression of aortic regurgitation in patients who undergo VSD closure without aortic valve surgery [14]. Our patient has therefore been referred for the repair of the aortic root with a VSD closure.

If our patient had not been diagnosed in time, he could have developed complications like worsening aortic regurgitation, permanent aortic insufficiency, dilatation of left heart chambers, congestive heart failure, or Eisenmenger syndrome. Our patient has therefore been referred for the repair of the aortic root with VSD closure. Following surgery, he can have complications like a progression of AI, residual shunt, arrhythmia, embolism, or infective endocarditis [13,15]. These have been well explained to the patient to make the final decision for his treatment.

## Conclusions

It is rare to find outlet VSDs with secondary aortic regurgitation in the elderly as most of them are likely to be detected at a younger age. However, it is essential to identify the cases as timely intervention can stop the progression and prevent permanent aortic insufficiency. Such patients, when identified, should be managed by VSD closure as well as aortic valve repair.
